# Understanding the role of key amino acids in regulation of proline dehydrogenase/proline oxidase (prodh/pox)-dependent apoptosis/autophagy as an approach to targeted cancer therapy

**DOI:** 10.1007/s11010-020-03685-y

**Published:** 2020-01-13

**Authors:** Thi Yen Ly Huynh, Ilona Zareba, Weronika Baszanowska, Sylwia Lewoniewska, Jerzy Palka

**Affiliations:** Department of Medicinal Chemistry, Faculty of Pharmacy, Medical University of Bialystok, 15-089, Bialystok, Poland

**Keywords:** Apoptosis, Autophagy, Proline dehydrogenase/proline oxidase, Proline, Glutamine

## Abstract

In stress conditions, as neoplastic transformation, amino acids serve not only as nutrients to maintain the cell survival but also as mediators of several regulatory pathways which are involved in apoptosis and autophagy. Especially, under glucose deprivation, in order to maintain the cell survival, proline and glutamine together with other glutamine-derived products such as glutamate, alpha-ketoglutarate, and ornithine serve as alternative sources of energy. They are substrates for production of pyrroline-5-carboxylate which is the product of conversion of proline by proline dehydrogenase/ proline oxidase (PRODH/POX) to produce ATP for protective autophagy or reactive oxygen species for apoptosis. Interconversion of proline, ornithine, and glutamate may therefore regulate PRODH/POX-dependent apoptosis/autophagy. The key amino acid is proline, circulating between mitochondria and cytoplasm in the proline cycle. This shuttle is known as proline cycle. It is coupled to pentose phosphate pathway producing nucleotides for DNA biosynthesis. PRODH/POX is also linked to p53 and AMP-activated protein kinase (AMPK)-dependent pathways. Proline availability for PRODH/POX-dependent apoptosis/autophagy is regulated at the level of collagen biosynthesis (proline utilizing process) and prolidase activity (proline supporting process). In this review, we suggest that amino acid metabolism linking TCA and Urea cycles affect PRODH/POX-dependent apoptosis/autophagy and the knowledge might be useful to targeted cancer therapy.

## Introduction

In stress conditions, cellular homeostasis is maintained by alteration of anabolic and catabolic processes. Anabolic processes are regulated by several factors affecting biosynthesis of cellular components. Major catabolic processes are mediated by the ubiquitin–proteasome system and autophagy [1]. In some cases, autophagy and apoptosis simultaneously occur in the same cell or autophagy precedes apoptosis via p53-dependent pathways or AMP-activated protein kinase (AMPK) [1]. Alternatively, autophagy can directly activate cell death pathway [1, 2]. Both p53 and AMPK are potent stimulators of proline dehydrogenase/proline oxidase (PRODH/POX) that has been implicated in the induction of autophagy and apoptosis [3–10]. Since PRODH/POX is linked to conversion of proline to pyrroline-5-carboxylate (P5C) [11], the availability of proline to this process is of critical importance. Proline and P5C are intermediates of interconversion of glutamine, glutamate, ornithine, and α-ketoglutarate suggesting the key role of these amino acids in the regulation of PRODH/POX-dependent apoptosis/autophagy. Therefore, this review aims to discuss the contribution of proline, glutamine, and its metabolites in regulation of PRODH/POX-dependent apoptosis/autophagy.

## Regulatory mechanism of autophagy and apoptosis

### Autophagy

Autophagy is a homeostatic, intracellular degradation process in which dispensable, long-lived, or aberrant proteins and damaged organelles are digested in lysosomes. The digestion products are recycled in cellular metabolism. It usually happens under stress conditions such as amino acid starvation [12–14]. Besides the removal of useless components retained in the cell, the other function of autophagy is to generate energy for synthesis of new building blocks in the process of homeostasis and cellular renovation [12, 13]. It suggests that autophagy has a profound impact on cancer cell survival [15]. Autophagy may also contribute to the suppression of cancer cell growth. The activation of autophagy explains a resistance mechanism in the course of cancer therapy. Therefore, the inhibition of autophagy was suggested as a potential pharmacotherapeutic approach for tumor growth suppression [13, 16].

A variety of proteins have been considered as autophagy markers for the assessment of presence or absence of autophagy in the cell. The first autophagy markers were found in yeast and identified more than 30 autophagy-related (ATG) genes, many of which have known orthologs in higher eukaryotes [17, 18]. Atg proteins have been classified into different groups based on their function in autophagy: (1) the Atg1/ULK complex (Atg1, Atg11, Atg13, Atg17, Atg29, and Atg31) regulates the induction of autophagosome formation; (2) the Atg9 complex (Atg2, Atg9, and Atg18), involved in membrane delivery to the expanding phagophore; (3) the PtdIns 3-kinase (PtdIns3K) complex (Vps34, Vps15, Vps30/Atg6, and Atg14) functions to recruit PtdIns3P-binding proteins; (4) two ubiquitin-like (Ubl) conjugation systems including the Atg12 complex (Atg5, Atg7, Atg10, Atg12, and Atg16) and a Atg8 complex (Atg3, Atg4, Atg7, and Atg8) that plays crucial role in vesicle expansion [19, 20] (Table 1). The mammalian ULK1/2 complex comprises ULK1/2 (mammalian homologs of Atg1), ATG13 (a homolog of yeast Atg13), RB1CC1/FIP200 (a putative Atg17 homolog), and C12orf44/ATG101 [21, 22]. The other study provided evidence that ULK1 kinase can be activated by AMP-activated protein kinase (AMPK) under glucose or amino acid starvation [23]. The ULK1/2 complex is inhibited by the phosphorylation of mTORC1 preventing interaction between ULK1 and AMPK. However, during induction of autophagy, the suppression of mTOR occurs and the protein complex of ULK1/2, ATG13, and RB1CC1 is formed to initiate the autophagy. Moreover, the autophagy process is mediated by Beclin-1 (autophagy-related gene, Atg 6) which codes for another autophagy protein [24, 25]. Some of these markers were linked to the PRODH/POX-dependent apoptosis/autophagy [3–10, 26, 27]. Since it has been proved that there is a cross-talk between autophagy and apoptosis [28], it cannot be excluded that the mechanism of this process may involve PRODH/POX.

**Table 1:** Classification of biomarkers of autophagy

ATG complex	Yeast	Mammals	Functions	References
Atg/ULK complex (regulates the class III phosphatidylinositol (PtdIns) 3-kinase complex)	Atg1	ULK1/2	Ser/Thr protein kinase; phosphorylated by M/TORC1; recruitment of Atg proteins to the PAS	[22]
	Atg13	ATG13	Regulatory subunit through phosphorylation by M/TORC1 and/or PKA, linker between Atg1 and Atg17	
	Atg17	RB1CC1/FIP200 (functional homolog)	Scaffold protein, ternary complex with Atg29 and Atg31. Phosphorylation by ULK1; scaffold for ULK1/2 and ATG13	
	C12orf44/Atg101		Component of the complex with ATG13 and RB1CC1	
Atg2-Atg18/Atg9 complex (maintenance of mitochondrial integrity)	Atg2	ATG2	Regulates Atg9 recycling from phagophore assembly site	[79]
	Atg18	WIPI1/2		
	Atg9	ATG9A/B	Required for autophagosome formation; Required for the efficient recruitment of Atg8 and Atg18	
	Atg23		Interaction with Atg9 Required for the biosynthetic cytoplasm to vacuole targeting (Cvt) pathway and efficient autophagy	[80]
PtdIns3K complex (Beclin1-Atg14-Ambra1- Vps15- Vps34)	Vps34	PIK3C3/VPS34	PtdIns 3-kinase	[18]
	Vps15	PIK3R4/VPS15	Ser/Thr protein kinase	
	Vps30/Atg6	BECN 1/Beclin 1	Component of PtdIns3K complex I and II Forms a complex with ER-associated Bcl-2 under nutrient-rich conditions and is released upon phosphorylation of Bcl-2 by JNK1	
		AMBRA1	Interacts with Beclin 1	
	Atg14	ATG14	Component of PtdIns3K complex I	
Atg8 complex (Ubiquitin-like conjugation system)	Atg8	LC3A/B/C, GABARAP, GABARAPL1/2	A unique ubiquitin-like conjugation to phosphatidylethanolamine on the autophagic membrane	[18, 81]
	Atg7	ATG7	E1-like enzyme	
	Atg3	ATG3	E2-like enzyme	
	Atg4	ATG4A-D	Cysteine proteinase LC3/Atg8 C-terminal hydrolase; deconjugating enzyme	
Atg12-Atg5-Atg16 Complex (Ubiquitin-like conjugation system)	Atg12	ATG12	Ubiquitin-like	[18]
	Atg7	ATG7	E1-like enzyme	
	Atg10	ATG10	E2-like enzyme	
	Atg16	ATG16L1	Activate Atg5; Interacts Atg12	
	Atg5	ATG5	Conjugated by Atg12 Directly binds membranes	

## Apoptosis

A concept of apoptosis was initially reported by Karl Vogt in 1872 then described by Walther Flemming who was the first to explain the mechanism of programmed cell death in 1885. Several studies suggested this mechanism as a program of cellular suicide where the cell destroys itself to maintain tissue homeostasis [29]. The machinery of apoptosis is mediated by a family of proteases, namely caspases which contain a cysteine at their active site and cleave the target proteins at a residue of aspartic acids [30]. Their precursors are called procaspases which are expressed as inactive forms in normal condition. These proteins, however, are cleaved to become active caspases triggering the apoptosis via energy-dependent cascade pathways [30]. The apoptosis is recruited through 3 different pathways: the extrinsic pathway, the intrinsic pathway, and Granzyme B-dependent pathway [31]. Among these pathways, the intrinsic and extrinsic pathways are the major mechanisms of apoptosis.

The intrinsic apoptosis pathway is activated by damages taking place within the cell. This mechanism involves the presence of pro-apoptotic proteins, BAX, and BID in the outer membrane of the mitochondria. They interact with the other protein, BAK to activate cytochrome c that binds to apoptotic protease activating factor-1 (Apaf-1) [32]. This binding activates active caspase 9 that triggers cascade downstream of effector caspases (such as caspase 3, caspase 7, and caspase 6), finally resulting in cell death [33]. The p53 protein is a key factor to activate the intrinsic pathway due to its contribution to activate BAX protein [34].

In contrast, the extrinsic pathway is initiated from extracellular events, triggered by ligand binding to plasma membrane death receptors, leading to activation of initiator caspase 8 [31]. Death receptors such as Fas/CD95 and tumor necrosis factor-related apoptosis inducing ligand (TRAIL) receptors DR-4 and DR-5 are transmembrane proteins that function to detect specific extracellular death signals [35, 36]. For instance, Adapter molecules like Fas Associated via Death Domain (FADD) contain death domain (DD) and a death effector domain (DED) which activate an active caspase-8 via a sequential action of a homotypic DED–DED interaction. Active caspase-8 generates a downstream of effector caspases contributing to cell death. However, they have the same execution pathway which is initiated by the activation of caspase-3 [31]. Typical biomarkers of apoptosis are listed in Table 2. Most of them were linked to PRODH/POX-dependent apoptosis [3–10].

**Table 2:** Typical biomarkers of apoptosis

Biomarker	Testing sample	Function	Method of detection	References
Activated caspase 2, 3, 7, 8 and 9	Tissue	Primary modulators of apoptosis	IHC, ELISA, flow cytometry, cytometric bead arrays	[82]
Caspase-3	Myocardial injury and cardiovascular disease	Responsible for chromatin condensation and DNA fragmentation	IHC, ELISA, flow cytometry, cytometric bead arrays	[82, 83]
Caspase 3/7	Hypothalamic cell model	Primary modulators of apoptosis	Multiplexing fluorescent and luminescent assays	[84]
Caspase 6	Neurodegenerative disorders (Alzheimer’s and Huntington disease)	Primary modulators of apoptosis	Electrochemiluminescence-based ELISA assay	[85]
Cytochrome C	Tissue, serum HL-60 cells and thymocytes	Transfer electrons from the cytochrome bc1 complex to cytochrome oxidase membrane	ELISA, flow cytometry	[82, 86]
CK18	Hepatocellular Carcinoma Treated with Sorafenib		M30- and M65-based sandwich ELISAs	[87]
Cytokeratins	Tissue, serum plasma		IHC, ELISA, flow cytometry,	[82]
Nucleosomal DNA	Tissue, serum		ELISA, DNA array, PCR	[82]
Apo-I/Fas, Fas ligand (sFAsL) Expressed on B and T cells as well as in normal and tumor tissue	Granulomatous disease	Increase the antigen-specific CD8( +) T-cell responses during viral infection	IHC, ELISA, flow cytometry	[82, 88, 89]
Bcl-2/Bcl-xl/Mcl-I	Cells, tissue		IHC, ELISA, flow cytometry	[82]
TRAIL	Inducing the autoimmune inflammation	Induces apoptosis through an extrinsic pathway,		[90]
Tumor protein p53 (TP53)	Colorectal cancer and other cancers	TP53 activation is capable of inducing apoptosis by intrinsic pathway	IHC, ELISA, flow cytometry	[82, 91]

*ELISA* enzyme-linked immunosorbent assay; *IHC* immunohistochemistry; *PCR* polymerase chain reaction

## PRODH/POX-dependent pathways relevant to apoptosis and autophagy

A variety of approaches to the inhibition of autophagy or activation of apoptosis have recently focused on proline dehydrogenase (PRODH), known also as proline oxidase (POX). PRODH/POX, a mitochondrial enzyme, converts proline to pyrroline-5-carboxylate (P5C) with the concomitant transfer of electrons to cytochrome c producing ATP or directly on oxygen generating reactive oxygen species (ROS) [5]. There are two human genes annotated as PRODH: PRODH1 (chromosome 22q11.21; NCBI Accession NM_016335) and PRODH2 (chromosome 19q13.12; NCBI Accession NM_021232). It has been suggested that the function of the enzyme may depend on substrate availability, proline. The main source of this amino acid is collagen which comprises 25% of total protein mass in animals [10, 30].

Briefly, these proteins are classified into major types which are type I in the skin, tendon, and bone, type II in cartilage, and type IV in basal laminae. Up to date, 28 types of collagen with 46 distinct polypeptide chains were found in vertebrates, as well as many other proteins containing collagenous domains [37, 38]. The predominant amino acids in collagen are proline and glycine, which enable triple-helical collagen structure. Extracellular degradation of collagens by tissue collagenases and further intracellular degradation of collagen degradation products in lysosomes release imidopeptides that are cleaved by cytoplasmic prolidase releasing a large amount of proline, the substrate for PRODH/POX.

After the conversion of proline to P5C, further proline metabolism is catalyzed by pyrroline-5-carboxylate dehydrogenase (P5CDH), transforming P5C into glutamate which is a precursor of α-ketoglutarate (α-KG) involved in the tricarboxylic acid (TCA) cycle. When the TCA cycle is overloaded by metabolites, the reversible reaction of conversion of P5C into proline by pyrroline-5-carboxylate reductase (P5CR) may occur, using NADPH or NADH as a cofactor. This interconversion of P5C-proline called proline cycle was first introduced in 1986 [39]. It has been demonstrated that the cellular proline, glutamine, and glutamate are linked to the proline pathway [40] regulating apoptosis/autophagy. The cycle is coupled to pentose phosphate shunt through NADPH from pentose pathway and NADP + from the proline cycle [4, 41]. Base on this mechanism, the role of PRODH/POX in the regulation of cellular metabolism has recently studied as an approach to cancer treatment. This cycle is responsible for the regulation of gene expression, purine biosynthesis, cellular redox state, apoptosis, and cell proliferation [3]. Moreover, PRODH/POX has a variety of regulatory functions, such as osmotic adjustment, protection against metabolic stress, and signaling in bacteria, plants, and mammals [10]. However, the most important function of PRODH/POX is donating electrons through flavin adenine dinucleotide (FAD) into the electron transport chain to generate ROS or ATP depending on environmental conditions [10].

## PRODH/POX-induced apoptosis

Both intrinsic and extrinsic pathways of apoptosis may be induced by PRODH/POX [42]. Especially, in the extrinsic pathway (death receptor), PRODH/POX stimulates the expression of tumor necrosis factor-related apoptosis-activated ligand (TRAIL), DR5, and cleavage of caspase-8 [42, 43], and also activates caspase-9 and caspase-3 [44, 45]. In cancer cells, PRODH/POX is upregulated by a variety of factors, for example tumor suppressor p53 and inflammatory factor peroxisome proliferator-activated receptor gamma (PPARγ) [7, 10]. However, its level in cancer tissue is much lower than that in normal tissues from the patients [46, 47]. Regarding the overexpression of POX, the ROS generation is integrated with the p53-dependent mechanisms [5, 48], switching the apoptotic cell death in a variety of cancer cell types [5, 48–51]. The supporting evidence showed that the PRODH/POX coding gene induced the expression of p53 [52]. On the other hand, inactivation of proline oxidase reduced p53-induced upregulation of proline oxidase, a release of cytochrome c from mitochondria, and apoptosis in cancer cells [42, 49]. PRODH/POX acting as a driver of apoptosis was clearly evaluated in a model of PRODH/POX knockdown cancer cells [53].

## PRODH/POX-induced autophagy

The recent study of Zareba et al., (2018) showed that in knocked down PRODH/POX MCF-7 breast cancer cells, cytoplasmic proline accumulation induced autophagy. However it was established that environmental conditions such as hypoxia or glucose deficiency may affect PRODH/POX-dependent autophagy/apoptosis [9]. It seems that proline availability may determine PRODH/POX-dependent apoptosis/autophagy. Although the mechanism of this process is not known, it has been suggested that hypoxia-inducible factor-1 alpha (HIF-1*α*) plays an important role in cancer cell metabolism. The availability of proline in the cell facilitates generation of *α*-KG that inhibits the transcriptional activity of HIF-1*α*. An increase in αKG concentration leads to an increase in the activity of a prolyl hydroxylase domain (PHD) of HIF-1*α* inducing proteasomal degradation of HIF-1*α* [43, 45, 54]. In contrast, proline through the same mechanism inhibits the activity of PHD, contributing to a decrease in HIF-1α proteasomal degradation and increase in its transcriptional activity.

It is well established that glutamine and proline metabolism, as well as other non-essential amino acids, are involved in oncometabolism of cells [9]. This process is called as “parametabolic pathway”. Particularly, the proline biosynthetic pathway was linked to glucose metabolism and POX-dependent apoptosis that is under the regulation of oncogene MYC.

Depending on the metabolic situation, proline can either be used for protein synthesis or oxidized in the mitochondria for energy production. Under nutrient deficiency and hypoxia, cancer cells may adopt to switch a survival mechanism which is the degradation of proline to produce the energy [26]. Therefore, hypoxia, glucose depletion, or treatment with rapamycin stimulated degradation of proline and POX-dependent autophagy.

## The impact of amino acids on cell re-programming

Several amino acids have been linked to activation or inhibition of apoptosis/autophagy [55]. It is well recognized that they participate in the mTORC1 and GCN2/eIF2 pathways which function to regulate protein translation and control the cellular demand for amino acids by concomitantly regulating autophagy-dependent catabolism [56–58]. For instance, non-essential amino acids (NEA) as proline in condition of glucose deprivation activate anti-apoptotic pathways in cancer cells by inducing the expression of anti-apoptotic members of the Bcl-2 gene family and preventing the expression of pro-apoptotic proteins [59]. The study suggested that although under low glucose condition apoptosis could be induced in cancer cells, the non-essential amino acids may counteract the process. It was supported by the upregulation of amino acid transporter gene LAT1 in the membranes of cancer cells [27, 60, 61] under glucose stress [59].

Glutamine was proved to be a sustainable source of energy. Early findings indicated that tumor formation is significantly due to the mitochondrial vulnerability through the alteration of glycolysis [62]. The proliferation of cancer cells is mostly maintained by energy products derived from the TCA cycle [63, 64]. A larger majority of tumor suppressors and oncogenes have been linked to metabolic pathways [64–67]. Glutamine is an integral metabolite in the proliferation of mammalian cells. The consumption rate of glutamine in cancer cells is compared to that of other amino acids. However, the demand for glutamine was observed to be tenfold higher than that for other amino acids [68]. Glutamine has profound impact on the functional activity of mammalian target of rapamycin (mTOR) kinase, mitochondrial membrane potential, and NADPH production [69]. Glutamine is a nitrogen source both for purine and pyrimidine synthesis [70, 71]. In the non-essential amino acid synthetic pathways, glutamine-derived glutamic acid continues donating its amine group to accelerate the tricarboxylic acid (TCA) cycle metabolites for the production of α-ketoglutarate, serine, alanine, aspartate, and ornithine. Glutamine acts as a source of carbon and nitrogen for the synthesis of proline, ornithine, and arginine as well as a donor for the synthesis of asparagine from aspartic acid [69]. Lack of exogenous glutamine is one of the major causes for the death of cancer cells [72]. Several tumor cell lines, generated from pancreatic cancer, glioblastoma multiforme, acute myelogenous leukemia, and small cell lung cancer, are substantially vulnerable due to glutamine starvation [73]. The study suggested that derivatives of glutamine like glutamate, α-ketoglutarate, and glutathione are involved in the apoptotic pathway [74]. Similarly, proline interconvertibility with glutamate and arginine [3, 75] may play an important role in cell programming. However, recent data linked glutamine metabolism and apoptosis/autophagy through P5C to urea cycle.

**Fig. 1:**
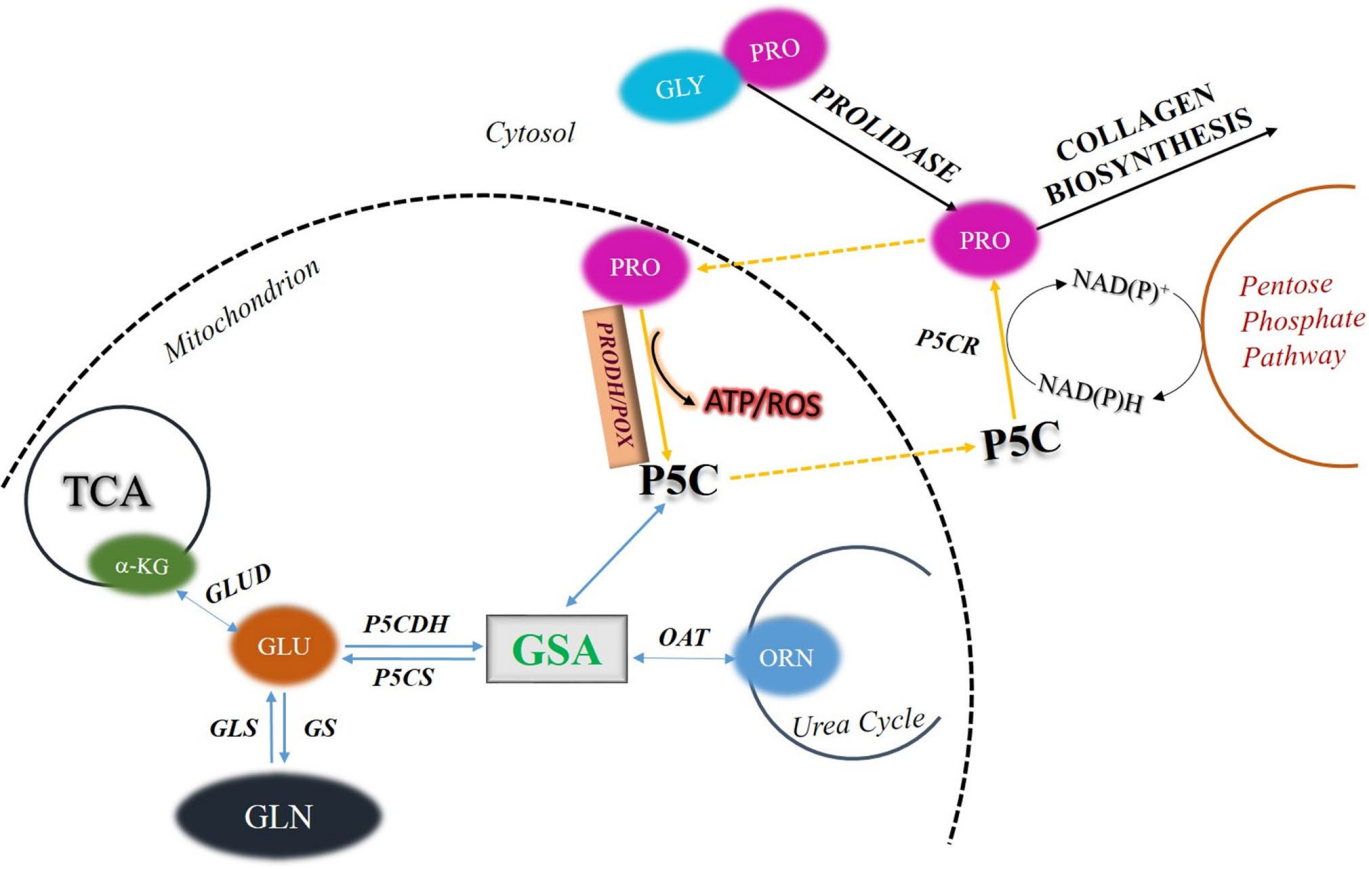
Regulation of PRODH/POX-dependent apoptosis/autophagy by key amino acids. *PRO* proline; *GLU* glutamate; *ORN* ornithine; *GLN* glutamine; *GLYPRO* glycyl-proline; *PRODH/POX* proline dehydrogenase (PRODH)/proline oxidase (POX); *ROS* reactive oxygen species; *P5C* pyrroline-5-carboxylate; *P5CR* pyrroline-5-carboxylate reductase; *P5CDH* pyrroline-5-carboxylate dehydrogenase; *P5CS* pyrroline-5-carboxylate synthase; *OAT* ornithine aminotransferase; *GSA* glutamic gamma-semialdehyde; *αKG α-*ketoglutarate; *TCA* tricarboxylic acid cycle; *GS* glutamine synthase; *GLS* glutaminase; *GLUD* glutamate dehydrogenase

Ornithine and glutamate are important sources of P5C. Ornithine is converted into P5C in a reaction catalyzed by mitochondrial vitamin B6-dependent ornithine-*δ*-aminotransferase (OAT), while glutamate through a reduction reaction catalyzed by mitochondrial ATP- and NAD(P)H-dependent P5C synthase (P5CS) [76, 77]. This reaction can be reversed by mitochondrial P5C dehydrogenase (P5CDH) [76]. The role of this metabolic pathway in apoptosis/ autophagy was supported by data showing that degradation of ornithine by ornithine decarboxylase (ODC) play an important role in cell proliferation, differentiation, and cell death. It has been demonstrated that decreasing the activity of ODC by difluoromethylornithine (DFMO) causes accumulation of intracellular reactive oxygen species (ROS) and cell arrest, thus inducing cell death. These findings indicate that urea cycle contributes to the regulation of apoptosis and autophagy [78]. Since ornithine is easily convertible into P5C (products of catalytic activity of PRODH/POX), it may affect PRODH/POX-dependent apoptosis/autophagy. The results of these studies allow us to present a hypothesis on the regulation of PRODH/POX-dependent apoptosis/autophagy by key amino acids (Fig. 1). During conversion of PRO into P5C by PRODH/POX, ATP or ROS is generated inducing autophagy or apoptosis. PRO availability for this process is critical requirement for PRODH/POX-dependent function. PRO comes from collagen degradation products (last step of the degradation is catalyzed by prolidase) or proline convertible amino acids, mainly GLU and ORN. Conversion of PRO into P5C takes place in mitochondria, while P5C into PRO mainly in cytoplasm. This process is known as a “proline cycle” and is coupled to pentose phosphate pathway generating nucleotides for DNA biosynthesis. Interconversion of PRO, GLU, and ORN through intermediate GSA to P5C may represent an interface regulating PRODH/POX-dependent P5C generation and ATP/ROS for autophagy/apoptosis. The process links TCA and Urea cycles to proline cycle providing complex regulatory mechanism of PRODH/POX-dependent functions. Understanding the interplay between key amino acids and TCA/Urea metabolites and their role in the regulation of PRODH/POX-dependent apoptosis/autophagy might be a promising approach to targeted cancer therapy.

## Conclusion

Studies of last decade provided several lines of evidence for the regulatory role of proline availability in PRODH/POX-dependent apoptosis/autophagy in cancer cells. The enzyme expression is often downregulated in various tumors, limiting mitochondrial proline degradation and PRODH/POX-dependent apoptosis. Critical factor for the process is proline availability that depends on the activity of prolidase (enzyme supporting cytoplasmic proline level) and the rate of proline utilization in the process of collagen biosynthesis. However, proline also represents an energy-sensing molecule that reprograms cellular metabolism. Interconversion of proline, glutamate, and ornithine links TCA cycle, urea cycle, and amino acid metabolism to PRODH/POX-dependent apoptosis/autophagy. Deregulation of energetic metabolism in cancer cells due to Warburg’s effect facilitates protein degradation as an alternative source of energy. Therefore, when glucose supply is limited, cancer cells may select proline as an alternative energy source. Therefore, amino acid metabolism in specific environmental cellular conditions may represent interface of PRODH/POX-dependent apoptosis and autophagy. The hypothesis is outlined in Fig. 1.
